# Modulating HIV-1 replication by RNA interference directed against human transcription elongation factor SPT5

**DOI:** 10.1186/1742-4690-1-46

**Published:** 2004-12-27

**Authors:** Yueh-Hsin Ping, Chia-ying Chu, Hong Cao, Jean-Marc Jacque, Mario Stevenson, Tariq M Rana

**Affiliations:** 1 Department of Biochemistry and Molecular Pharmacology, University of Massachusetts Medical School, 364 Plantation Street, Worcester, MA 01605, USA; 2 Program in Molecular Medicine, University of Massachusetts Medical School, 373 Plantation Street, Worcester, MA 01605, USA; 3 Department and Institute of Pharmacology National Yang-Ming University Shih-Pai, Taipei 11221 Taiwan

## Abstract

**Background:**

Several cellular positive and negative elongation factors are involved in regulating RNA polymerase II processivity during transcription elongation in human cells. In recruiting several of these regulatory factors to the 5' long terminal repeat (LTR) promoter during transcription elongation, HIV-1 modulates replication of its genome in a process mediated by the virus-encoded transactivator Tat. One particular cellular regulatory factor, DSIF subunit human SPT5 (hSpt5), has been implicated in both positively and negatively regulating transcriptional elongation but its role in Tat transactivation *in vivo *and in HIV-1 replication has not been completely elucidated.

**Results:**

To understand the *in vivo *function of hSpt5 and define its role in Tat transactivation and HIV-1 replication, we used RNA interference (RNAi) to specifically knockdown hSpt5 expression by degrading hSpt5 mRNA. Short-interfering RNA (siRNA) designed to target hSpt5 for RNAi successfully resulted in knockdown of both hSpt5 mRNA and protein levels, and did not significantly affect cell viability. In contrast to hSpt5 knockdown, siRNA-mediated silencing of human mRNA capping enzyme, a functionally important hSpt5-interacting cellular protein, was lethal and showed a significant increase in cell death over the course of the knockdown experiment. In addition, hSpt5 knockdown led to significant decreases in Tat transactivation and inhibited HIV-1 replication, indicating that hSpt5 was required for mediating Tat transactivation and HIV-1 replication.

**Conclusions:**

The findings presented here showed that hSpt5 is a *bona fide *positive regulator of Tat transactivation and HIV-1 replication *in vivo*. These results also suggest that hSpt5 function in transcription regulation and mRNA capping is essential for a subset of cellular and viral genes and may not be required for global gene expression.

## Background

The elongation phase of transcription is often a critical juncture for regulating gene expression [1,2] and a number of genes including c-myc, c-fms, hsp70, and those encoded by HIV-1 are regulated at this stage of transcription [3-6]. During transcription elongation, shortly after successful initiation of RNA synthesis, RNA polymerase II (RNA pol II) can pause, arrest, pass through terminator sequences, or terminate transcription. The varying processivity of RNA pol II prior to entering productive elongation is controlled by the action of both negative and positive transcription elongation factors (N-TEFs and P-TEFs, respectively). The function of P-TEFs is to reduce the barrier of N-TEFs and promote the release of RNA pol II from the transition state that can cause termination of transcription [7]. Three elongation regulatory factors, P-TEFb (positive transcription elongation factor b), DSIF (DRB (5,6-*d*ichloro-1-β-D-*r*ibofuranosyl*b*enzimidazole) sensitivity-inducing factor) and NELF (negative elongation factor), have been identified using DRB as a transcription inhibitor [8-10] and function together to regulate transcription elongation.

Modulation of HIV-1 gene expression provides one fundamental example of how transcription elongation can be controlled by such regulatory factors [11-14]. Tat, an HIV-1 regulatory protein, is required for synthesis of viral mRNA and increases the efficiency of transcription elongation from the HIV-1 promoter. In the presence of Tat, the processivity of RNA Pol II complexes that initiate transcription in the HIV-1 5' long terminal repeat (5' LTR) region becomes greatly enhanced. For this increased processivity to occur, Tat binds with a nascent leader RNA element, *trans*-activation responsive (TAR) RNA, located at the 5' end of all HIV-1 transcripts [15]. Cellular factors in association with Tat and TAR are then recruited to the 5' LTR, stimulating RNA pol II processivity during elongation. More specifically, the C-terminal domain (CTD) of RNA pol II is proposed to be hyperphosphorylated by P-TEFb during Tat transactivation to promote elongation [12-14]. Composed of cyclin-dependent kinase CDK9 and Cyclin T1, P-TEFb has been shown to bind the activation domain of Tat and TAR RNA loop sequence and phosphorylate the CTD of RNA pol II [16-18]. Tat transactivation is postulated to involve Tat-TAR interactions that then give rise to the recruitment of P-TEFb to RNA pol II complexes at the 5' LTR. This recruitment is necessary to enhance the processivity of RNA Pol II from the HIV-1 5' LTR promoter [7,14,17,19]. Thus, TAR RNA provides a scaffold for Tat and P-TEFb to bind and assemble a regulatory switch during HIV replication [20].

Human DSIF consists of subunits hSpt5 and hSPT4 and was originally discovered as a negative elongation factor that binds to RNA pol II [9]. In conjunction with NELF, DSIF represses transcriptional elongation at positions proximal to promoters [9,10]. Escape from transcriptional repression imposed by DSIF and NELF requires P-TEFb, which has been shown *in vitro *to phosphorylate both hSpt5 and CTD [7,10,21-29]. Interestingly, hSpt5 is conserved among eukaryotes and is a dual transcriptional regulator that can function as both a negative and positive elongation factor [30-32]. Currently, it is postulated that phosphorylation of hSpt5 and RNA pol II by P-TEFb is the key event during which hSpt5 functionally switches from a negative barrier to a positive elongation factor during transcription in human cells. Methylation of SPT5 also has been shown to regulate its interaction with RNA pol II and this posttranslational modification of SPT5 may alter transcriptional elongation functions in response to viral and cellular factors [33].

Although hSpt5's role in transcription regulation in association with P-TEFb has been established, its involvement in Tat transactivation and HIV-1 replication continues to be elucidated. Several *in vitro *studies have shown that hSpt5 is required for Tat transactivation and that both hSpt5 and RNA pol II phosphorylation is stimulated after recruitment of P-TEFb by Tat [25,29,34]. hSpt5 may also play a positive role in Tat transactivation through its association with human mRNA capping enzyme (HCE), which is a bifunctional triphosphatase-guanylyltransferase required for capping mRNA (reviewed in [1,35]), since SPT5, Tat, and CTD associate with the capping apparatus to stimulate capping [36-43]. However, studies in a recent report have suggested that only P-TEFb hyperphosphorylation of the RNA pol II CTD is directly required for Tat transactivation, precluding a direct role for hSpt5 in RNA pol II processivity during HIV-1 replication [26]. Therefore, hSpt5 role in Tat transactivation and HIV-1 replication *in vivo *remains unclear.

Here, we used RNA interference (RNAi) to address whether hSpt5 is required for Tat transactivation and thus HIV-1 replication *in vivo *and further defined hSpt5 cellular functions. RNAi is a remarkably efficient process whereby double-stranded RNA (dsRNA) induces sequence-specific degradation of homologous mRNA in animal and plant cells (reviewed in ref. [44]). In mammalian cells, RNAi can be triggered by 21-nucleotide (nt) small interfering RNA (siRNA) duplexes and a few dsRNA molecules are sufficient to inactivate a continuously transcribed target mRNA for an observable period of time [45,46]. RNAi has recently been used to successfully knockdown the expression of a number of HIV genes, including p24, reverse transcriptase, vif, nef, tat, and rev, and has led to pre- and post-integrative HIV-1 RNA degradation and reduced HIV infectivity [47-52]. These results suggested that targeting viral factors required for the HIV life cycle with siRNAs including those required for HIV replication is a viable method for treating HIV infections. Other groups have targeted cellular factors implicated in supporting the HIV life cycle, including T-cell co-receptors CD4, CXCR4, CCR5, and CD8 [50,52-54] and transcription factor NF-κB [51], which has a role in HIV transcription initiation. Knockdown of the co-receptors reduced HIV infectivity, effectively blocking HIV entry into cells [55]. RNAi has become one of the leading methodologies for studying gene knockdown in human cells. During RNAi, a double-stranded 21-nucleotide (nt) short-interfering RNA (siRNA) targets a specific, complementary mRNA for degradation, resulting in significantly decreased expression, or knockdown, of the targeted gene (reviewed in [56,57]). In this report, siRNA designed to target hSpt5 successfully silenced hSpt5 as observed by decreased hSpt5 mRNA and protein expression. In addition, RNAi directed against hSpt5 did not significantly affect cell viability. hSpt5 knockdown led to significant decreases in Tat transactivation and inhibited HIV replication, indicating that hSpt5 was required for Tat transactivation and HIV replication *in vivo*. Taken together, silencing of hSpt5 by RNAi firmly established that the regulation of HIV-1 gene expression requires both Tat-TAR-P-TEFb interactions and interactions between RNA pol II transcription complexes and hSpt5.

## Results

### Specific silencing of hSpt5 expression by siRNA in HeLa cells

**Figure 1:**
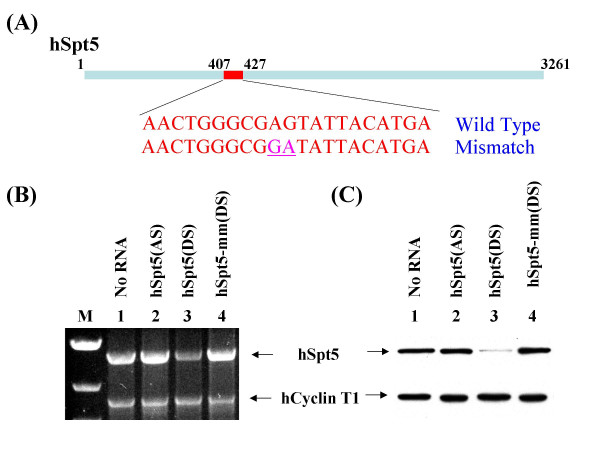
**Specific silencing of hSpt5 expression by RNAi**. (A) hSpt5 mRNA is 3261 nucleotides in length. siRNA targeting sequence for hSpt5 was selected from position 407 to 427 relative to the start codon. As a specific control, mutant siRNA containing 2 nucleotide mismatches (underline) between the target mRNA and the antisense of siRNA at the hypothetical cleavage sites of the mRNA was generated. (B) Evaluation of specific hSpt5 siRNA activity by RT-PCR. Total cellular mRNA was prepared from HeLa cells transfected without siRNA or with hSpt5 duplex or control siRNAs and was followed by RT-PCR, as described in Material and Methods. Each RT-PCR reaction included 100 ng total cellular mRNA, gene-specific primer sets for hSpt5 and hCycT1 amplification (0.5 μM for each primer), 200 μM dNTP, 1.2 mM MgSO_4 _and 1U of RT/platinum *Taq *mix. Primer sets for hSpt5 produced 2.6 kb products while hCycT1 produced 1.8 kb products. RT-PCR products were resolved on a 1% agarose gel and viewed by ethidium bromide staining. RT-PCR products are shown from cells that were not transfected with siRNA (lane 1), or cells transfected with single-stranded antisense hSpt5 siRNA (hSpt5 (AS), lane 2), hSpt5 duplex siRNA (hSpt5 (DS), lane 3), or mismatch hSpt5 duplex siRNA (hSpt5-mm (DS), lane 4). Lane M is a marker lane. (C) Analysis of specific hSpt5 siRNA activity by western blotting. Cell lysates were prepared from HeLa cells mock-transfected without siRNA (lane 1), or transfected with single-stranded antisense hSpt5 siRNA (hSpt5 (AS), lane 2), hSpt5 duplex siRNA (hSpt5 (DS), lane 3), or mismatch hSpt5 duplex siRNA (hSpt5-mm (DS), lane 4). Cell lysates were analyzed by 10% SDS-PAGE. Protein contents were detected by immunoblotting assay with antibodies against hSpt5 (top panel) and hCycT1 (lower panel).

To inhibit hSpt5 expression in a cultured human cell line using RNAi, siRNA targeting an hSpt5 sequence from position 407 to 427 relative to the start codon was designed (Figure 1A). Magi cells were transfected with this hSpt5 duplex siRNA using Lipofectamine (Invitrogen). To evaluate the effects of hSpt5 RNAi, total cell lysates were prepared from siRNA-treated cells harvested at various time points after transfection. hSpt5 mRNA or protein levels were analyzed by RT-PCR or western blot using anti-hSpt5 antibodies, respectively. Cells transfected with hSpt5 siRNA had significantly lowered hSpt5 mRNA (Figure 1B, lane 3) and protein expression (Figure 1C, lane 3), indicating that siRNA-mediated silencing of hSpt5 had occurred successfully. hSpt5 knockdown was consistently between ~85–90%. This knockdown effect was dependent on the presence of the 21-nt siRNA duplex harboring a sequence complementary to the mRNA target. As shown in Figures 1B and 1C, mock-treated (no siRNA) (lane 1), single-stranded antisense hSpt5 siRNA (lane 2), or mismatched hSpt5 duplex siRNA (lane 4) containing two nucleotide mismatches between the target mRNA and siRNA antisense strand at the putative cleavage site of the target mRNA (Figure 1A) did not affect hSpt5 mRNA or protein levels. These results showed that hSpt5 knockdown was specific to duplex siRNA exactly complementary to the hSpt5 mRNA target. In evaluating either mRNA or protein levels, human Cyclin T1 (hCycT1) was used as an internal control, showing that the effects of hSpt5 siRNA were specific to hSpt5 and did not effect hCycT1 mRNA or protein expression (Figure 1B and 1C, lower panel). Taken together, these results demonstrated that hSpt5 knockdown was sequence specific and led to significantly decreased hSpt5 mRNA and protein levels.

### Kinetics of hSpt5 knockdown by RNAi

**Figure 2:**
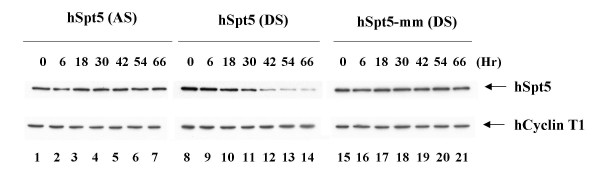
**Kinetics of specific hSpt5 siRNA activity by Western blotting**. HeLa cells were transfected with single-stranded antisense hSpt5 siRNA (hSpt5 (AS), lanes 1–7), hSpt5 duplex siRNA (hSpt5 (DS), lanes 8–14), or mismatch hSpt5 duplex siRNA (hSpt5-mm (DS), lanes 15–21) having 2 nucleotide mismatches between the target mRNA and the antisense strand of siRNA at the hypothetical cleavage site of the mRNA. Cells were harvested at various times post transfection. Protein content was resolved on 10% SDS-PAGE, transferred onto PVDF membranes, and immunoblotted with antibodies against hSpt5 (top bands) and hCycT1 as an internal control (lower bands).

Having established that hSpt5 could be knocked down using RNAi, the kinetics of hSpt5 knockdown were examined. To perform kinetic experiments, hSpt5 duplex siRNA, single-stranded antisense hSpt5 siRNA, or mismatch duplex hSpt5 siRNA were transfected into Magi cells. Cell lysates were collected at various time points to assay for protein levels during hSpt5 knockdown. Immunoblot analysis using anti-hSpt5 antibodies revealed the timing of gene suppression and persistence of hSpt5 RNAi effects in Magi cells during the time course experiment (Figure 2). hSpt5 knockdown was first observed between 30–42 h post-transfection, with maximum knockdown (~85–90% knockdown) occurring at 42–66 h post transfection (Figure 2, lane 8–14). Protein levels gradually recovered to normal levels between 66–90 h (data not shown), indicating that the effects of hSpt5 siRNA did not last indefinitely. Neither single-stranded antisense siRNA (Figure 2, lanes 1–7) nor mismatched duplex siRNA (Figure 2, lanes 15–21) affected hSpt5 protein levels throughout the duration of the time course. These results indicated that hSpt5 knockdown by RNAi occurred after 30 h and these knockdown effects were specific to duplex siRNA targeting hSpt5.

### Knockdown of hSpt5 is not lethal to human cells

**Figure 3:**
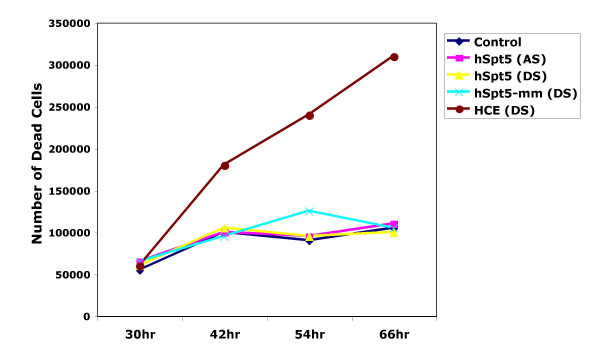
**Analysis of cell viability by counting trypan blue-stained cells**. HeLa cells were transfected with Lipofectamine with various siRNAs or no siRNA. Three siRNA duplexes, including hSpt5 siRNA (yellow), mismatch hSpt5 siRNA (light blue) and siRNA targeting human capping enzyme (HCE, red), were used in these experiments. Controls for viability included cells mock-transfected with no siRNA (dark blue) or cells transfected with single-stranded antisense hSpt5 siRNA (purple). At various times after transfection, cells floating in the medium were collected and counted in the presence of 0.2% trypan blue. Cells that took up dye (stained blue) were counted as not viable.

Knowing that the kinetics of hSpt5 peaked at 42–54 h post-transfection, we were able to evaluate the viability of cells during hSpt5 knockdown experiments over varied time intervals. Cell viability was assessed using trypan blue exclusion at various times after a single transfection of various siRNAs. As shown in Figure 3, during the 66 h time course experiment, the number of non-viable hSpt5 knockdown cells (yellow line) observed was comparable to mock-treated cells (no siRNA; dark blue line). Cells transfected with single-stranded antisense hSpt5 siRNA (purple line) or mismatched hSpt5 duplex siRNA (light blue line) that did not show hSpt5 knockdown also showed minimal changes in cell viability.

hSpt5 has been shown to interact with the human mRNA capping enzyme (HCE) and this interaction enhances capping enzyme guanylylation and mRNA capping several fold [40]. Since Spt5, Tat, and CTD associate with the capping apparatus to stimulate capping [36,37,39-42], we planned to separately define the role of HCE and hSpt5 in Tat transactivation by using RNAi to specifically knockdown HCE expression. HCE knockdown was confirmed by RT-PCR (data not shown). In contrast to hSpt5 knockdown cells, HCE knockdown cells showed a significant increase in cell death (Figure 3, red line) over the course of the knockdown experiment. These results indicated that HCE is an essential enzyme for cell viability and growth. Simialr findings showing that RNA capping was essential for metazoan viability have also been previously reported using RNAi in *C. elegans *[58]. These results indicated that hSpt5 knockdown was not lethal to human cells, while a much more stringent requirement for HCE expression was essential for cell viability.

### Role of hSpt5 on HIV-1 Tat Transactivation

To examine whether hSpt5 was required for HIV-1 Tat transactivation *in vivo*, Tat transactivation during hSpt5 knockdown in Magi cells was monitored. Magi cells are a HeLa cell line harboring a stably integrated single copy of the HIV-1 5' LTR-β-galactosidase gene. These cells are also genetically programmed to express the CD4 receptor for HIV-1 infection ([59]; see below). In this experiment, Magi cells were co-transfected with Tat expression plasmid pTat-RFP and hSpt5 duplex siRNA. Co-transfection with Tat siRNA was used as a positive control for inhibition of Tat transactivation while single-stranded antisense hSpt5 siRNA and mismatched siRNA were used as negative controls. Tat transactivation and protein levels were evaluated by harvesting cells 48 h post transfection, which was within the timeframe that hSpt5 knockdown peaked. Expression of HIV-1 Tat-RFP under the control of the CMV early promoter was confirmed by western blot using anti-RFP antibody and by measuring RFP fluorescence using a fluorescence spectrophotometer (data not shown). In addition, immunoblot analysis confirmed that hSpt5 siRNA specifically inhibited hSpt5 protein expression in the absence and presence of HIV-1 Tat protein in Magi cells (data not shown).

**Figure 4:**
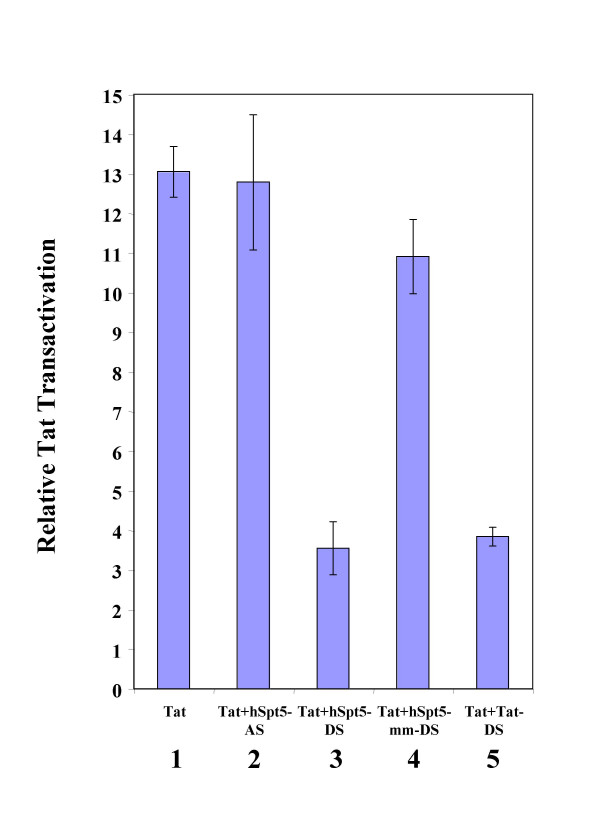
**Effect of hSpt5 siRNA on HIV-1 Tat transactivation in Magi cells**. Quantified effect of siRNA on HIV-1 Tat transactivation was determined by measuring β-galactosidase activity. Magi cells were co-transfected with pTat-RFP plasmid and various siRNAs targeting hSpt5 or Tat and harvested at 48 h post-transfection. Activity of β-galactosidase was measured using the β-Galactosidase Enzyme Assay System (Promega). Tat transactivation was determined by the ratio of β-galactosidase activity in pTat-RFP transfected cells to activity measured in cells without pTat-RFP. The inhibitory effect of siRNA was determined by normalizing Tat transactivation activity to the amount of Tat-RFP protein. Tat transactivation was measured for Magi cells transfected with pTat-RFP only (lane 1), or Tat-RFP transfected with single-stranded antisense hSpt5 siRNA (hSpt5-AS, lane 2), hSpt5 duplex siRNA (hSpt5-DS, lane 3), mismatch hSpt5 duplex siRNA (hSpt5-mm-DS, lane 4), or Tat siRNA duplex (Tat-DS, lane 5). Results are representative of three independent experiments.

Tat-RFP enhances the expression of genes that are under the control of the HIV-1 5' LTR promoter. In this experiment, Tat transactivation was measured by assaying the β-galactosidase activity resulting from expression of the β-galactosidase gene under the HIV-1 5' LTR promoter. To quantify the effects of various siRNAs on HIV-1 Tat transactivation, the ratio between β-galactosidase activity in cells transfected with pTat-RFP (with or without siRNAs) and mock-treated cells not transfected with pTat-RFP was determined. The results of this quantitation are shown in Figure 4. In Magi cells, Tat-RFP strongly stimulates the expression of β-galactosidase, represented by a 13-fold increase in Tat transactivation (Figure 4, lane 1). On the other hand, Tat transactivation was strongly inhibited in cells transfected with Tat siRNA (~90% knockdown; Figure 4, lane 5), as previously shown [51]. Tat transactivation was similarly inhibited when cells were transfected with hSpt5 duplex siRNA, exhibiting only ~30% of the Tat transactivation observed with Tat-RFP alone (Figure 4, lane 3). Neither antisense hSpt5 siRNA nor mismatched hSpt5 siRNA (Figure 4, lane 4) showed any effect on Tat transactivation. These results indicated hSpt5 knockdown caused by siRNA specifically targeting hSpt5 mRNA inhibited HIV-1 Tat transactivation in human cells. These results strongly supported an important role for hSpt5 in Tat transactivation *in vivo *and suggested that RNAi of hSpt5 had the potential to inhibit HIV-1 replication.

### hSpt5 knockdown inhibits HIV-1 replication

To evaluate the effect of hSpt5 knockdown on HIV-1 replication, a double siRNA transfection protocol was used to maximize the knockdown efficiency of hSpt5 during HIV-1 infection. Magi cells were transfected with siRNA directed against hSpt5. Cells mock transfected without siRNA, or transfected with single-stranded antisense hSpt5 siRNA or mismatch hSpt5 siRNA were used as negative controls. Transfection with Vif or Nef siRNAs was used as a positive control [20]. 24 h after the first transfection, a second siRNA transfection identical to the first was performed. 24 h later, doubly transfected cells were infected with various concentrations of HIV_NL-GFP_, an infectious molecular clone of HIV-1. Knockdown of hSpt5 protein levels was then evaluated 48 h post infection in doubly transfected cells. An even larger decrease in hSpt5 protein levels was observed in doubly transfected cells (~95% knockdown) as compared to singly transfected cells (~85–90% knockdown; Supplementary Figure 1, compare lanes 4 and 10), suggesting that more robust knockdown of gene expression can be achieved using this double transfection method.

**Figure 5:**
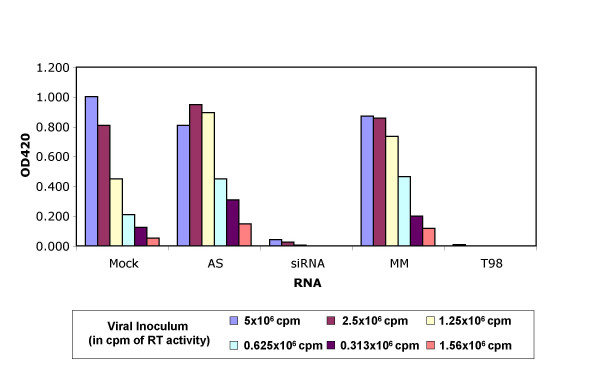
**siRNA targeting hSpt5 modulate HIV-1 replication**. HeLa-CD4-LTR/β-galactosidase (Magi) cells were mock-transfected (mock), or transfected with single-stranded antisense hSpt5 siRNA (AS), hSpt5 duplex siRNA (siRNA), mismatched hSpt5 duplex siRNA (MM) or Vif duplex siRNA (T98). 24 h after the first transfection, a second siRNA transfection was performed. 24 h later, cells were infected with HIV_NL-GFP_, an infectious molecular clone of HIV-1. Cells infected with virus and not treated with oligofectamine are shown (mock). HIV-1 Tat-mediated transactivation of the 5' LTR led to β-galactosidase production, which was quantified 48 h post-infection. Cells treated with duplex siRNA targeting Vif (lanes marked T98 [47]) served as a positive control. Serial double dilutions of the viral inoculum (in cpm of RT activity) are consistent with 32-fold decreases in viral replication.

HIV-1 Tat-mediated transactivation of the 5' LTR occurring in cells infected with virus led to β-galactosidase production, which was also quantified 48 h post-infection. In this single-cycle replication assay for evaluating HIV-1 replication, β-gal activity reflected the activity of reverse transcriptase and viral replication of varying amounts of viral inoculum. Therefore, changes in β-gal activity could be directly correlated to changes in the efficacy of HIV-1 replication. The positive siRNA control targeting HIV-1 Vif showed decreased levels of β-gal activity and HIV-1 replication, as shown previously (Figure 5; [47]). Double-stranded siRNA directed against hSpt5 showed a similar decrease in β-gal activity when compared with Vif knockdown. This observed decrease was equivalent to the β-gal activity measured when using 32 times less viral inoculum with mock-treated cells (Figure 5), indicating that hSpt5 knockdown had significantly reduced HIV-1 replication. p24 levels were also monitored during these experiments and decreased in the context of hSpt5 knockdown (data not shown), supporting the conclusion that hSpt5 knockdown has a negative effect on the HIV-1 life cycle. Control experiments using hSpt5 single-stranded antisense or mismatched duplex siRNA duplexes showed no antiviral activities. In addition, no significant toxicity or cell death was observed during these experiments, suggesting that hSpt5 knockdown was not lethal even in the context of HIV-1 infection. These results demonstrated that hSpt5 silencing using RNAi modulated HIV-1 replication and firmly established an important role for hSpt5 in Tat transactivation and HIV-1 replication *in vivo*.

## Discussion

hSpt5, as part of the DSIF complex, was originally discovered as a negative elongation factor required for conferring DRB sensitivity to transcription elongation complexes thereby inhibiting transcription [9]. This negative barrier provided by hSpt5 was thought to be relieved through P-TEFb phosphorylation of both hSpt5 and RNA pol II CTD, which results in increased processivity of RNA pol II complexes [7,10,21-29]. Increased processivity has also been linked to the phosphorylated form of hSpt5 conferring a positive effect on transcription elongation [25,29,34]. Recently, however, it has been shown that Tat is able to enhance transcription elongation *in vitro *in the absence of hSpt5 [26]. These results appeared to indicate that P-TEFb phosphorylation of RNA pol II was the sole event that directly led to Tat transactivation and increased RNA pol II processivity [26]. Thus, from the results of all of these *in vitro *studies collectively, the requirement for hSpt5 in positively regulating transcription elongation during Tat transactivation has remained unclear.

Here, we studied the role of hSpt5 *in vivo *using RNAi and established that hSpt5 played a positive role in Tat transactivation and HIV-1 replication. Knockdown of hSpt5 provided insight into several functional aspects of the hSpt5 protein. First, knockdown of hSpt5 was not lethal in Magi cells, indicating that hSpt5 was not required for cell viability. This was an interesting result because studies of SPT5 mutants in yeast and zebrafish and RNAi of SPT5 in *C. elegans *have shown that SPT5 was essential for growth and/or embryonic development in those organisms [30,31,60]. It seems likely that hSpt5 holds similar essential functions in human cells during embryonic development but may not be absolutely required in adult cells. Alternatively, hSpt5 knockdown may have led to decreased levels of expression that were still sufficient for hSpt5 to carry out its essential functions. Our results support the notion of using RNAi against hSpt5 as a potential therapeutic strategy for fighting HIV-1 infection since there is the potential that HIV-1 functions could be targeted for inhibition without significantly interfering with host cell functions.

**Figure 6:**
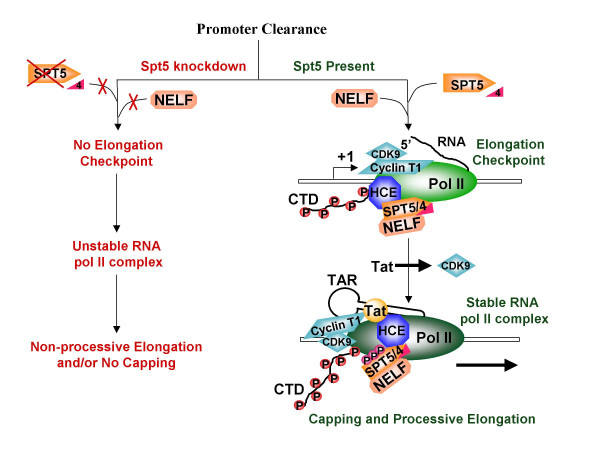
**Model for Tat transactivation in absence or presence of SPT5**. See text for details.

The key finding of this study was that hSpt5 knockdown significantly inhibited both Tat transactivation and HIV-1 replication. These results indicated that hSpt5 was a *bona fide *regulator of Tat transactivation that is required for HIV-1 replication *in vivo*. Our *in vivo *results strongly support previous *in vitro *results recapitulating Tat transactivation that showed immunodepletion of hSpt5 significantly inhibited Tat transactivation [29,34]. However, it is difficult to reconcile our *in vivo *results with recently published *in vitro *experiments showing that P-TEFb hyperphosphorylation of the CTD in the absence of hSpt5 still enhanced RNA pol II processivity during Tat transactivation [26]. In reconciling whether P-TEFb hyperphosphorylation was directly required for Tat transactivation to the exclusion of hSpt5, we would like to propose that the required function of P-TEFb hyperphosphorylation may be distinct from the role hSpt5 plays in enhancing RNA pol II processivity during Tat transactivation. In our model (Figure 6), P-TEFb hyperphosphorylation would occur first, triggering enhanced processivity of RNA pol II. hSpt5 presumably is phosphorylated at around the same time as RNA pol II, stimulating hSpt5 to switch from a negative regulator to a positive elongation factor [25]. Phosphorylated hSpt5 may then be important for positively regulating an initial step in Tat transactivation.

Conceivably, hSpt5 functions in transcription elongation as a stabilization factor that enhances the stability of RNA pol II elongation complexes formed after P-TEFb hyperphosphorylation of the CTD. This type of role would also support hSpt5 function as an antiterminator factor as described previously [61]. Another important positive function for hSpt5 during Tat transactivation may involve hSpt5 and Tat interactions with the capping machinery [40-42]. Phosphorylation of hSpt5 by P-TEFb may stabilize hSpt5 interactions with HCE thereby stabilizing Tat and CTD interactions with the capping machinery to promote capping and successful production of stable HIV transcripts (see model in Figure 6). Due to the highly structured nature of TAR, capping of the 5' end of HIV transcripts is not very efficient in the absence of Tat [41,42] and Tat stimulated capping may require the presence of hSpt5 for greater access to the 5' end or to stabilize and kinetically arrest the elongation complex. Capping of HIV transcripts has also been shown to occur more proficiently when elongation is paused and not continuous [42], suggesting that DSIF/NELF-dependent pausing of early stage elongation complexes is representative of an elongation checkpoint. One function of this checkpoint may be to allow time for the recruitment of capping machinery and subsequent capping of HIV RNA to stabilize nascent transcripts prior to further elongation. In the absence of hSpt5, pausing may no longer occur during elongation since neither NELF nor hSPT4 binds to RNA pol II without hSpt5 [62,63]. Thus, the window for the capping apparatus to be recruited by Tat and/or stimulate capping may be severely shortened or lost altogether without hSpt5. Any resulting uncapped HIV transcripts would be prone to degradation, accounting for the lower level of Tat transactivation and HIV replication observed during hSpt5 knockdown. The potential roles for phosphorylated hSpt5 in stabilizing RNA pol II processive elongation complexes or with respect to capping during Tat transactivation are not mutually exclusive as shown in Figure 6. hSpt5 may indeed have multi-functional roles as a positive regulator during HIV-1 replication.

## Conclusions

The *in vitro *and *in vivo *approaches taken to address the importance of hSpt5 function all shed light on the multi-faceted nature of Tat transactivation. Accordingly, these studies altogether support important roles for both P-TEFb and hSpt5 in mediating transcription elongation during HIV-1 replication *in vivo*. The dual function of hSpt5 as a negative and positive transcription elongation factor also demonstrates the complexity associated with transcriptional regulation during transcription elongation and HIV-1 Tat transactivation. It is likely that posttranslational modifications of hSpt5 dictate functions of Spt5 at various promoters. Further studies will be required to elucidate how various modifications of hSpt5 such as phosphorylation and methylation control transcription elongation of both cellular and viral genes.

## Methods

### siRNA preparation

Twenty-one nucleotide siRNAs were chemically synthesized as 2' bis(acetoxyethoxy)-methyl ether-protected oligos (Dharmacon, Lafayette, CO). Synthetic RNAs were deprotected, annealed and purified using standard protocols provided by the manufacturer. Formation of duplex RNA was confirmed by 20% non-denaturing polyacrylamide gel electrophoresis (PAGE). Sequences of siRNA duplexes were designed as described previously [46] and subjected to a BLAST search against the NCBI EST library to ensure that only the desired genes were targeted.

### Culture and transfection of cells

Magi (multinucleate activation of galactosidase indicator) cells carrying the endogenous HIV-5'LTR β-galactosidase gene were maintained at 37°C in Dulbecco's modified Eagle's medium (DMEM, Invitrogen) supplemented with 10% fetal bovine serum (FBS), 0.2 mg/ml of Geneticin (G418) and 0.1 mg/ml Hygromycin B (Roche Molecular Biochemicals). Cells were passaged at sub-confluence and plated at 70% confluency for transfection. Reporter plasmids and siRNA were co-transfected into Magi cells using Lipofectamine (Invitrogen) in duplicate 6-well plates (Falcon). A transfection mixture containing 25–150 nM siRNA and 9 μl of Lipofectamine in 1 ml of serum-reduced OPTI-MEM (Invitrogen) was added to each well. For high efficiency knockdown experiments, 150 nM siRNA was used. After incubating at 37°C for 6 h, cells were cultured in antibiotic-free DMEM. For further analysis, transfected cells were harvested at various time intervals, washed twice with phosphate buffered saline (PBS, Invitrogen), flash frozen in liquid nitrogen, and stored at -80°C.

### RT-PCR for amplification of hSpt5 and hCycT1 mRNA

Total cellular mRNA was prepared from HeLa cells transfected without siRNA or with hSpt5 or control siRNAs using a Qiagen RNA mini kit, followed by an oligotex mRNA mini kit (Qiagen). RT-PCR was performed using a SuperScript One-Step RT-PCR kit with platinum *Taq *(Invitrogen) and 40 cycles of amplification. Each RT-PCR reaction included 100 ng total cellular mRNA, gene-specific primer sets for hSpt5 and hCycT1 amplification (0.5 μM for each primer), 200 μM dNTP, 1.2 mM MgSO_4 _and 1U of RT/platinum *Taq *mix. Primer sets for hSpt5 produced 2.6 kb products while hCycT1 produced 1.8 kb products. RT-PCR products were resolved on a 1% agarose gel and viewed by ethidium bromide staining. Forward and reverse primer sequence for amplifying SPT5 were 5'-ATGTCGGACAGCGAGGACAGC-3' (nts 1–21) and 5'-TGTACATGGCCGGCGTCCC-3' (nts 2638–2656), respectively. Forward and reverse primer sequences for amplifying hCycT1 are 5'-GCAACAAGTTCAAGATCTGGTCAT-3' (nts 381–404) and 5'-CCCGGGGGATCCTTACTTAGGAAGGGGTGGAAGTGG-3' (nts 2158–2200); underlined sequences represent restriction enzyme sites), respectively.

### Western blotting

siRNA treated cells were harvested as described above and lysed in 1X reporter lysis buffer (Promega). After centrifugation to remove cellular debris, concentrations of proteins in lysates were determined using a Dc protein assay kit (Bio-Rad). Proteins in 30 μg of total cell lysates were fractionated by 10% SDS-PAGE, transferred onto a polyvinylidene difluoride membrane (PVDF membrane, Bio-Rad), and immunoblotted with antibodies against hSpt5 (Pharmingen) and hCycT1 (Santa Cruz Biotech). Protein content was visualized by a BM chemiluminescence Blotting Kit (Roche Molecular Biochemicals). The blots were exposed to X-ray film (Kodak MR-1) for various times (between 1 s and 5 min).

### β-galactosidase enzyme assay

Magi cells were harvested 48 h after transfection with Tat-RFP plasmids in the absence or presence of siRNAs. Cell lysates were prepared and quantified as described above. To perform standard β-galactosidase assays, 120 μg of cell lysates were mixed in 150 μl of 1X reporter lysis buffer and 150 μl of 2X β-galactosidase assay buffer (Promega), and incubated at 37°C for 30 min. To stop the reaction, 500 μl of 1 M sodium carbonate was added to the mixture and mixed well by vortexing briefly. Absorbance of the reaction mixture was read immediately at 420 nm. The amount of Tat-RFP protein was determined using a fluorescence spectrophotometer (Photon Technology International). 300 μg of cell lysates was subjected to the spectrophotometer with slit widths set at 4 nm for both excitation and emission wavelengths as described previously [46,64]. Fluorescence of Tat-RFP in the cell lysate was detected by exciting at 558 nm and recording the emission spectrum from 568 nm to 650 nm; the spectrum peak at 583 nm represents the maximum fluorescence intensity of Tat-RFP. Tat transactivation was determined by calculating the ratio of β-galactosidase activity (absorbance at 420 nm) of the pTat-RFP transfected cells to that of cells without pTat-RFP plasmid treatment. The inhibitory effect of siRNA treatment was determined by normalizing Tat-transactivation activity to the amount of Tat-RFP protein (represented by RFP fluorescence intensity) in the presence and absence of siRNA.

### Magi infectivity assay

HeLa-CD4-LTR/β-gal indicator (Magi) cells [59] were plated in 24-well plates (7.5 × 10^5 ^cells per well) and transfected with siRNAs as previously described [47]. siRNA (60 pmol) was transfected into cells using oligofectamine (2 μl, Invitrogen) for 3 h in serum-free DMEM (GIBCO). Cells were rinsed twice and top-layered in 500 μl of DMEM-10% FBS. 24 h after the first transfection, a second identical siRNA transfection was performed. 24 h after the second transfection, cells were trypsinized and seeded in 96-well microtiter plates (4 × 10^4 ^cells per well), incubated 3 h and infected with HIV_NL-GFP_, an infectious molecular clone of HIV-1. HIV-1 virions (normalized to RT activity in cpm) were added in doubling dilutions to duplicate wells. 48 h post infection, cells were harvested to quantify β-galactosidase activity and protein levels.

## Competing Interests

The author(s) declare that they have no competing interests.

## Authors' contribution

Y-HP carried out all the Spt5 silencing experiments, C-yC performed capping enzyme knockdown experiments, HC performed quantitative analysis of the data, J-MJ performed HIV-1 replication assays, and MS analyzed and interpreted HIV inhibition results. TMR conceived the ideas, and participated in the experimental design and in drafting the manuscript.
